# The Combination of Physical Activity and Cognitive Games is Associated With Better Cognitive Performance and Gray Matter Volume in Older Adults

**DOI:** 10.1002/gps.70121

**Published:** 2025-07-03

**Authors:** Ben Rattray, Joseph M. Northey, Disa J. Pryor, Allison A. M. Bielak, Kaarin J. Anstey, Nicolas Cherbuin

**Affiliations:** ^1^ Discipline of Sport and Exercise Science Faculty of Health University of Canberra Canberra Australia; ^2^ National Centre for Epidemiology and Population Health Australian National University Canberra Australia; ^3^ Department of Human Development and Family Studies Colorado State University Fort Collins Colorado USA; ^4^ School of Psychology University of New South Wales Sydney Australia; ^5^ Neuroscience Research Australia Randwick Australia

**Keywords:** brain, cognitive training, exercise, lifestyle, MRI

## Abstract

**Objectives:**

Investigate whether engaging in cognitive and physical activity is associated with cognitive performance and gray matter volume. Specifically, this study investigated the role of both activity types performed in close temporal proximity to each other.

**Methods:**

Cognitively healthy older adults (*n* = 155; 73–78 years; 45% female) enrolled in the PATH Through Life study with valid cognitive, MRI and physical activity (PA) measures were included in the study. PA was objectively measured with a SenseWear Armband for 7 days. PA and cognitive engagement were self‐reported through a 1‐week activity diary. The number of 3‐h periods in which ≥ 15 min of objective moderately vigorous physical activity (MVPA) > 3METs, cognitive activity, or both occurred, were assessed. Cognitive game activity periods were also coded. Associations between activity engagement and outcome measures were assessed with hierarchical regression models while controlling for age, sex, and education.

**Results:**

For cognitive engagement, greater *activity diversity* was associated with better symbol digits modalities test performance (SDMT), while a greater number of *cognitive activity periods* were associated with better SDMT, and digit span backward performance. Greater *cognitive game periods* improved model fit for several cognitive outcomes and right hippocampal volume. *MVPA periods* were not associated with any outcome. The number of periods in which *cognitive activity and MVPA* were present together was associated with better immediate recall. Periods in which *cognitive games and MVPA* co‐existed were associated with cognitive and volumetric outcomes.

**Discussion:**

These findings support the notion that both cognitive and physical activity are positively related to brain health. They highlight the potential importance of performing both activity types in close temporal proximity to support the aging brain.

## Summary

- Periods in which cognitive games and MVPA co‐existed were associated with the widest range of cognitive and volumetric outcomes.
- Cognitive games were associated with cognitive and volumetric outcomes to a greater extent than cognitive activity not considered “games”.
- MVPA by itself, assessed as the number of periods with at least 15 min MVPA, was not associated with any outcome.

## 1 Introduction

Moderate to strong evidence for 14 modifiable risk factors for dementia, which collectively account for about 45% of all cases, has been demonstrated [1]. Amongst them, a lack of cognitive and physical activity is commonly linked to increased rates of cognitive decline [1]. Since limiting neuropathological damage and increasing or maintaining cognitive reserve are two demonstrated ways of preventing dementia, it is important to understand the roles of cognitive and physical activity in achieving this. Recent evidence suggests that cognitive and physical activity performed together may provide more cognitive benefits than when each activity is performed alone [2]. Despite this, little is understood about how habitual engagement with these activities, particularly when executed in close temporal proximity, are related to cognitive health within naturalistic settings.

Multiple mechanisms and pathways are thought to contribute to the development of cognitive reserve [3]. Early life education is thought to contribute to developing an individual's cognitive reserve, which may then be preserved across adulthood through ongoing engagement in cognitively stimulating activities. e.g., some of the variability in cognitive performance during middle‐age appears to relate to engagement in stimulating cognitive activities, even after accounting for education [4]. Lee and colleagues [5]. observed that adults who participated in cognitively engaging activity in their 70s recorded lower rates of dementia over a median 5‐year follow‐up. Similar findings have been observed between cognitive activity levels and cognitive performance [6, 7], although this may not extend to supporting overall brain volume [7]. A recent meta‐analysis however suggests that both social and cognitive activities display a small effect size with several volumetric MRI brain measures [8]. Thus, it may be that cognitive activity mediates the association between social activity and cognitive performance [9].

Cognitive games may represent a type of cognitive activity in which competitive engagement, social interaction and/or individual preferences further enhance the benefits of this type of activity. e.g., engagement in Sudoku and similar puzzles has been positively associated with cognitive outcomes [10]. and an intervention focused on board and card games in older adults has been shown to support cognitive functions [11]. Within the public domain at least, various commercial enterprises market “brain games” for cognitive benefits, although a scientific consensus on their effectiveness remains elusive [12].

In another domain, physical activity also appears to promote brain health through both protection against neurodegeneration and by supporting a cognitive reserve. Indeed, several meta‐analyses of short‐term physical activity interventions support its role in improving, or at least protecting, cognitive function in older adults [13, 14]. However, self‐reported physical activity appears to be more closely associated with baseline cognitive performance, which is then maintained, rather than changes in cognitive performance over time [15]. Within older populations, objectively assessed physical activity is positively associated with brain structure [16, 17], which may account for improved preservation of cognitive function. Even a minor difference in physical activity (i.e., a 3–5 min mean difference of moderate‐vigorous physical activity between Quartile 1 and Quartile 2) appears to lower the risk of cognitive impairment by as much as ∼36% [18], but the evidence is not consistent. Although a larger cross‐sectional study (*n* = 6452) demonstrated a correlation between physical activity and cognitive outcomes [18], smaller studies (*n* = 167–262) [16, 17] did not. Collectively, this may indicate that physical activity alone is not sufficiently potent to result in measurable benefits to cognitive health in many settings. Physical activity, though, can result in large but transient increases in brain‐derived neurotrophic factor (BDNF) [19], alongside other physiological changes, including elevated blood flow. This transient increase may provide the conditions in which the brain can consolidate networks through BDNF‐supported long‐term potentiation. It is plausible then that the conjunction of active cognitive and physical lifestyles provides superior support for the maintenance of cognitive function, particularly if they occur in close temporal proximity.

This hypothesis appears to be supported by the effectiveness of multi‐modal interventions that target both cognitive and physical training to support cognitive function in older adults [2, 20]. These combined interventions appear to provide additional benefits compared to cognitive [21] or physical training [20, 22] alone. Few observational studies have investigated these relationships. A recent study within the UK Biobank has, however, suggested that greater adherence to both physical and cognitive activities—the latter represented by frequent friend/family visits which are typically considered social engagement ‐ are associated with a lower risk of dementia [23]. Further, in a relatively small study of older adults (*n* = 43, mean 72 years), self‐reports of both cognitive and physical activity have been positively associated with gray matter volumes in frontal areas, with common associations within the anterior part of the hippocampus (Arenaza‐Urquijo et al., 2017). These investigations have not investigated whether engagement in these activities occurred together. Regardless, the rich neuroplastic environment created by physical activity [24] offers an opportunity for cognitive pathways to be consolidated with the addition of cognitive stimulation. Over time, if this behavior is repeated often enough, then improvements or retention in both cognitive function and gray matter volume could be anticipated. This relationship between cognitive and physical activity, performed together, requires investigation within observational settings.

This study aimed to address this research gap by investigating the associations between engagement in physical and cognitive activities, cognitive performance and brain volumes in a large population of generally healthy community‐living older individuals. We hypothesized that the frequency of cognitively engaging activity reported in the same 3‐h period as at least 15 min of moderate‐vigorous physical activity (MVPA) would be associated with cognitive performance and regional gray matter volume in a cohort of older adults.

## 2 Methods

### 2.1 Study Population and Design

Participants were drawn from the magnetic resonance imaging (MRI) sub‐study [25] of the Personality and Total Health through life (PATH) project, which is described in detail elsewhere [26]. Briefly, in 2001, the older (60–64 years at baseline) PATH study cohort was randomly drawn from the electoral roll (registration on the electoral roll is compulsory for Australian citizens) to produce a wave 1 sample of 2551 (from a response rate of 58%). Of those, 622 randomly selected participants were offered, and 478 eventually underwent a structural MRI scan. At the fourth assessment (∼12 years after baseline, 73–78 years), 275 participants underwent a repeat MRI scan. At the MRI scan appointment, these individuals were invited to participate in a cross‐sectional sub‐study examining the cognitive, physical, and dietary habits of older adults [27]. Of those, 184 participants accepted and completed the current sub‐study. Participants with MRI images of poor quality or invalid MRI data, who wore a SenseWear Armband (SWA; BodyMedia, PA, USA) for less than five valid days (> 20 h on‐body time), had a history of neurological disorders (stroke, Parkinson's disease, epilepsy or dementia), or scored ≤ 25 on the Mini‐Mental State Examination, were excluded, leaving 165 participants available for inclusion. Further, several individual diaries through which cognitive activity was sampled were deemed incomplete, or unsuitable for coding (e.g., deemed insufficient information completed, hand‐writing illegible) and were excluded (*n* = 11) from the analysis. This left a final study sample (*n* = 155, 45% female) that did not differ from the larger PATH study cohort in terms of BMI, sex, and completed years of education, although it was slightly younger (75.1 vs. 75.6 years; *p* < 0.001; Appendix A1). The study was approved by the Australian National University Human Research Ethics Committee (#2010/542 and #2012/703), and participants provided written informed consent.

### 2.2 Measures

#### 2.2.1 Cognitive Activity Engagement

Participants were provided with a blank 7‐day paper‐based diary, divided into 3‐h time periods between 6 a.m. and 12 midnight, and a 6‐h block between 12 midnight and 6 a.m. Participants were instructed to list the activities they engaged in during each block, as well as their duration. Participants were free to choose the activities they listed, although they were informed that the research team were investigating which activities may link to a person's memory, reasoning, and attention. The examples provided to participants were: “Read newspaper”; “Went to exercise class at a senior center”; “Read emails”; “Watched TV”, and; “attending a Yoga class”. Participants were encouraged to record activity immediately, within the 3‐h block, or at least on the same day the activity was undertaken. Participants were instructed to leave a day blank if they missed it. Blocks left blank were considered as containing no activity. Previously, similar daily activity diaries have been linked to cognitive function in older adults [28, 29].

Two researchers (BR and JN) independently coded the daily diaries. Initially, 15 diaries were coded in pilot work to compare coding and gain a consensus on categories. The coding of activities was further discussed and refined with AB. Subsequently, the coding of activities fell into one of 32 coded activities (Table 1).

**TABLE 1:** Categories of activity coded from the daily diaries.

Theme	Activities coded
Physical	Resistance training Aerobic training (walking, run, swim, bike) Flexibility exercise (yoga, tai chi) Outdoor recreational (fishing) Physical games (tennis, bowling, golf) Gardening/household maintenance Household chores (cooking, laundry, cleaning)
Daily living	Self‐care (shower, get dressed) Driving/public transport Shopping Attend appointment ^a^Child care ^a^Provide care/transport to spouse/dependent (not child care) ^a^Work (at a job)
Games	^a^Solitary games (crossword, Sudoku or computer games) ^a^Social games (cards, board games)
Social	Interact in person with friends/family/acquaintances Talk on phone (or Skype) Play with pets ^a^Attending club meeting/mentoring/teaching
Cultural/Hobby	Cultural activity (play, concert, museum, library, sporting event) Attending religious service ^a^Educational activity (in person or online tutorial) ^a^Artistic pursuit (Crafts, art, photography, music)
Non‐physical activities	Watch TV/Movies Listen to radio ^a^Reading (leisure or newspaper) ^a^Writing, not email (e.g. document, stories, letters) ^a^Using computer (not games) ^a^Pray/meditate ^a^Reminiscing/thinking ^a^Organizing (holiday, event), researching or home admin (Finances, mail)

^a^ Activities included as cognitive activity in the analysis.

Activity coding originally included the duration of each activity, but as many participants did not record the duration of activities they were simply coded as being present within each period. Activities that obviously overlapped time periods were coded as existing in both. Activities had to be specifically mentioned, not implied (e.g. travel could not be coded as driving unless it was explicit), to be coded.

Out of the 32 activity categories coded, 14 were subjectively considered to require a higher level of cognitive effort and were coded as “cognitive activities” (Note “a” in Table 1). This decision was based on the anticipated higher‐order executive and planning functions associated with these tasks. Two of these activities (solitary games and social games) were considered separately as “cognitive games” given that cognitive, or “brain games”, are often marketed to improve cognitive health and typically involve higher cognitive skills, although a consensus on their effectiveness remains elusive [12]. Cognitive activity periods and cognitive game periods were exclusive and did not overlap in activity‐coded content. In addition, physical activities that were deemed to have a higher cognitive component (typically sports with a skill component—e.g., bowls, golf, tennis) were also included as “cognitive activities” (not “cognitive games”). “Activity diversity”, reflecting the number of different activities engaged in over the entire diary, was recorded within the coding process, as this has been shown to predict cognitive ability [30]. The activity types recorded for analysis are presented in Table 2.

**TABLE 2:** Activity coding types.

Variable	Description
Activity diversity	The number of different activities coded within an individual's activity diary across the week (32 activity types could be coded per day)
Cognitive activity periods	The number of 3‐h periods in which cognitive activities were coded. Sum over the 7 days.
Cognitive game periods	The number of 3‐h periods in which cognitive games were coded. Sum over the 7 days.
MVPA periods	The number of 3‐h periods in the SWA recorded physical activity > 3 METs for at least 15 min. Sum over the 7 days.
Cognitive activity and MVPA periods	The number of 3‐h periods in which cognitive activity and MVPA periods were recorded together. Sum over the 7 days.
Cognitive games and MVPA periods	The number of 3‐h periods in which cognitive games and MVPA periods were recorded together. Sum over the 7 days.

*Note:* There were up to 6 maximum time periods per day.

Abbreviations: MVPA = moderate‐vigorous physical activity; SWA = SenseWear armband.

#### 2.2.2 Objective Physical Activity

Participants were fitted with a SWA on the day of their MRI scan and wore it over the triceps muscle of their left arm for a continuous 7‐day period to objectively record PA. The SWA incorporates a tri‐axial accelerometer with galvanic skin response, skin temperature, near‐body ambient temperature, and heat flux to noninvasively measure PA [31] with greater accuracy than accelerometry alone [32]. The SWA was set to record data at 1‐min intervals and was only removed during water submersion. Data from the SWA were downloaded to the proprietary software (SenseWear Professional version 8.0, BodyMedia, PA) where energy expenditure was calculated [31]. For this analysis, we only investigated moderate to vigorous intensity PA (> 3 METs; MVPA) as this is what is most commonly associated with brain health outcomes in the broader literature [13]. Further, to determine which 3‐h periods included a notable duration of MVPA, we utilized a minimum threshold of 15 min of MVPA recorded by the SWA within each 3‐h period. This duration of MVPA was nominally chosen as 15 min as this amount has been suggested to be the minimum daily requirement for health benefit [33].

#### 2.2.3 Cognitive and Physical Activity

Utilizing the cognitive and physical activity thresholds described, we summed the number of 3‐h periods in which both cognitively engaging activity and MVPA occurred together. The coding variables are included in Table 2.

#### 2.2.4 MRI Data Acquisition

As previously described [17], 3‐dimensional structural MRI were obtained on a Siemens 1.5 T Espree scanner (Siemens Medical solutions). The T1‐weighted MRI was acquired in sagittal orientation using an MPRAGE sequence with the following parameters: repetition time, echo time, flip angle and slice thickness equal to 1160 ms/4.24 ms/15° and 1 mm, respectively, with matrix size 512 x 512 for a final voxel size of 1 x 0.5 × 0.5 mm [34].

#### 2.2.5 Image Processing

Image processing included intensity and inhomogeneity correction, skull stripping, tissue segmentation, and parcellation according to the FreeSurfer atlas [35]. Utilizing the FreeSurfer 5.3 [36] cross‐sectional pipeline, processing included automated segmentation and parcellation to delineate regions of interest (ROIs) and estimation of cortical surfaces and cortical thickness for each participant. Quality control was implemented via an in‐house script that identified outliers based on total gray and white matter volumes. Visual inspection of the data occurred post‐FreeSurfer processing with any outliers removed from the analysis.

#### 2.2.6 Regions of Interest

Total brain volume and regional volumes of the dorsolateral prefrontal cortex (DLPFC) and hippocampus were selected a priori for analysis due to their sensitivity to physical activity interventions [37] and their role in mediating the relationship between aerobic fitness and cognitive function [38]. Further, cognitive activity engagement has been previously associated with hippocampal and frontal cortex volumes [8]. In the current study, DLPFC volume was computed by summing the volume of the superior frontal, rostral mid‐frontal and caudal mid‐frontal gyri [17].

#### 2.2.7 Cognitive Assessment

The Symbol Digit Modalities Test (SDMT)[39] and the Trail Making Test Part B (TMT‐B)[40] were used to assess processing speed and executive function, respectively. Additionally, processing speed was independently assessed using the Trail Making Test Part A (TMT‐A)[40]. Verbal working memory was assessed using the Digits‐Span Backwards Task, a sub‐test of the Weschler memory scale [41]. Episodic memory was assessed with the first list of the California Verbal Learning Test for both immediate and delayed recall [42]. For these cognitive assessments, a higher score indicates better performance, except for the TMT‐A and TMT‐B where a higher score reflects poorer performance.

#### 2.2.8 Health and Sociodemographic Covariates

Total years of education, smoking, alcohol consumption and depressive symptoms were assessed by self‐report during the fourth wave of standard assessment within the larger PATH study. Depressive symptomatology was assessed with the Goldberg Depression and Anxiety Scale [43].

Brachial blood pressure was measured in a seated position after resting for at least 5 min. Participants were classified as hypertensive if they had an average systolic blood pressure ≥ 140 mm Hg or diastolic blood pressure ≥ 90 mm Hg or were on anti‐hypertensive medication. Participants were considered diabetic if they reported having diabetes, were receiving drug treatment or consuming a special diet for diabetes, or if their fasting plasma glucose was ≥ 10 mmol/L [44] from a venous sample taken during the standard assessment within the larger PATH study. Body mass index was computed using the formula weight in kilograms divided by height in meters squared, where height and weight were measured by a trained anthropometrist. APOE*E4 genotype was determined from DNA collected by cheek swab on entry into the study [45].

### 2.3 Statistical Analysis

Statistical analysis was conducted with R version 4.3.1 [46]. To avoid potential collinearity with covariates, the residual method was applied to normalise volumetric regions of interest for intracranial volume (ICV) with the formula: adjusted volume = raw volume—*b* x (ICV—mean ICV), where *b* is the slope when regressing a region of interest volume on ICV [44]. Means and standard deviations were calculated for age, sociodemographic and health, the cognitive and physical activity variables, brain volume, and cognitive function variables. Students *t*‐tests for continuous and *Χ* ^2^ tests for categorical data were conducted to compare across sex.

For each activity measure (e.g., number of cognitive activity periods, periods in which cognitive and MVPA thresholds were met), hierarchical regression analyses were carried out in order to assess their unique contribution to predicting each outcome variable (volumetric or cognitive outcomes) above control variables. The initial nested model was constructed controlling for age, sex and education. Both age and education were mean‐centered within the models. A further model was then constructed using the original variables and adding the activity measure of interest. A significant improvement in the model fit between these two models was taken as evidence that the activity measure provided an improvement in the variance explained by the model. This process was repeated using a “fully controlled” model that additionally controlled for APOE*e4 genotype, hypertension, diabetes, smoking status, depression, alcohol consumption and body mass index (Appendix A2 and A3). The alpha level was set at *p* < 0.05.

Within all models, normality of residuals was tested via visual inspection of Q‐Q plots. During this process it became apparent that extreme outliers existed in the trail‐making tasks of the cognitive performance outcomes. One data point from the TMT‐A, and two from TMT‐B were removed after they were deemed to not be within the 75^th^ percentile, plus 3* the interquartile range. Z‐scores were recalculated for this variable with these scores removed. This was considered a conservative approach as without this step, significant differences existed between males and females for the TMT‐B time, and more variables were originally significant in the hierarchical analysis for both TMT‐A and ‐B test types.

## 3 Results

The demographic and health characteristics, and outcome variables, of the study sample are presented in Table 3. Few sex differences were identified. Males had somewhat higher years of education than females and higher DLPFC and total brain volumes. Males had higher (better) working memory scores on the backward digit task, but females had higher (better) immediate recall scores.

**TABLE 3:** Demographic and health characteristics of the study population.

Characteristics	All (*n* = 155)	Male (*n* = 86)	Female (*n* = 69)
Age, y (SD)	75.1 (1.3)	75.2 (1.4)	75.0 (1.3)
Range	73–78	73–78	73–78
BMI, kg·m^−2^ (SD)	26.2 (4.2)	26.8 (4.3)	25.5 (3.9)
Education, y (SD)	14.3 (2.7)	14.8 (2.6)	13.7 (2.7)^**^
Ever smoker, *n* (%)	63 (42)	42 (50)	21 (32)^*^
MMSE, score (SD)	29.2 (1.0)	29.1 (1.1)	29.3 (0.9)
Hypertension, *n* (%)	117 (75)	64 (74)	53 (77)
Diabetes, *n* (%)	21 (14)	14 (16)	7 (10)
APOE4 carrier*, *n* (%)	46 (30)	27 (31)	19 (28)
Activity variables, *n* (SD)			
Activity diversity	14.3 (3.4)	14.2 (3.1)	14.7 (3.7)
Cognitive activity periods	20.1 (8.5)	21.8 (8.3)	18.0 (8.4)**
Cognitive game periods	3.0 (4.3)	3.0 (4.7)	3.1 (3.6)
MVPA periods	8.1 (6.5)	9.8 (6.7)	6.0 (5.5)***
Cognitive activity and MVPA periods	3.8 (3.6)	5.1 (3.9)	2.3 (2.5)***
Cognitive games and MVPA periods	0.4 (1.0)	0.5 (1.1)	0.3 (0.8)
Regions of interest, mm^3^ (SD)			
L DLPFC	37,445 (4311)	39,355 (4076)	35,065 (3309)***
R DLPFC	36,833 (4270)	38,634 (4146)	34,588 (3254)***
L hippocampus	3851 (427)	3906 (473)	3782 (351)
R hippocampus	3816 (407)	3864 (437)	3756 (407)
Total brain volume	1,116,314 (116,309)	1,178,708 (105,637)	1,038,547 (75,017)***
Cognitive performance, SD			
SDMT, score	48.0 (8.1)	48.3 (8.0)	47.6 (8.2)
TMT‐A, sec	35.4 (10.3)	35.9 (10.5)	34.8 (10.1)
TMT‐B, sec	86.1 (32.8)	82.2 (29.7)	91.2 (36.0)
Digit backwards, words	5.2 (2.1)	5.5 (2.1)	4.8 (2.0)*
Immediate recall, words	5.5 (1.8)	5.2 (1.6)	5.9 (2.0)*
Delayed recall, words	7.8 (3.2)	7.4 (2.9)	8.3 (3.5)

*Note:* All region of interest volumes are corrected for intracranial volume.

Abbreviations: BMI, body mass index; DLPFC: dorsolateral pre‐frontal cortex; L, left; MMSE, Mini‐mental state examination; MVPA, moderate to vigorous physical activity; R, right; SDMT, Symbol Digits Modalities Test; TMT‐A, Trail Making Test A; TMT‐B, Trail Making Test B.

Significant difference between male and females denoted by:

^*^ *p* < 0.05.

^**^ *p* < 0.01.

^***^ *p* < 0.001.

Characteristics of the activity behavior are reported in Table 3. One female and one male recorded no periods in which any cognitive activities were reported. Periods in which cognitive games (as a subset of the cognitive activities more generally) were reported were lower, with 26 females (38%) and 47 males (55%), not reporting any cognitive game engagement. Physical activity, represented by periods in which a threshold of 15 min of MVPA was achieved, saw males record higher numbers of periods in which MVPA was reported. Ten females (15%), and six males (7%) did not report a period in which at least 15 min of MVPA was achieved. Males recorded a higher number of periods in which both cognitive activity and MVPA were reported. Twenty‐one females (30%), and 11 males (13%), did not record a single period in which cognitive activity and MVPA were recorded in the same period. Only 10 females (14%) and 19 males (22%) recorded at least one period in which cognitive game activity and MVPA was recorded.

### 3.1 Primary Outcomes

Cognitive activity variables were associated with several cognitive outcomes in hierarchical regression models controlling for age, sex, and education (Table 4). Greater *activity diversity* was associated with better performance on the SDMT, while a greater number of *cognitive activity periods* were associated with better SDMT and digit span backwards performance. Greater number of *cognitive game periods* were more frequently associated with cognitive and volumetric variables, improving model fit for SDMT, delayed recall, TMT‐A and TMT‐B, as well as right hippocampal volume.

**TABLE 4:** Contribution of activity diversity, cognitive activity periods, and cognitive game periods to cognitive and brain volume through a hierarchical model controlling for age, sex, and education.

	Activity diversity			Cognitive activity periods			Cognitive game periods		
	Δ adj. *R* ^2^	*p* of delta *R* ^2^	Beta	Δ adj. *R* ^2^	*p*	Beta	Δ adj. *R* ^2^	*p*	Beta
Cognitive performance									
SDMT	**0.030**	**0.015**	**0.194**	**0.025**	**0.026**	**0.197**	**0.079**	**< 0.001**	**0.290**
Immed. Recall	0.004	0.196	0.105	0.014	0.073	0.159	0.008	0.136	0.118
Delayed recall	−0.005	0.645	0.038	0.015	0.067	0.164	**0.022**	**0.035**	**0.167**
Digit span backwards	0.010	0.113	0.129	**0.021**	**0.039**	**0.184**	0.003	0.235	0.095
TMT‐A	0.016	0.064	−0.153	−0.002	0.404	−0.076	**0.033**	**0.014**	**−0.197**
TMT‐B	0.004	0.213	−0.107	0.015	0.071	−0.164	**0.051**	**0.003**	**−0.235**
Regions of interest									
L DLPFC	−0.003	0.568	0.041	−0.005	0.931	−0.007	0.004	0.173	0.095
R DLPFC	−0.002	0.403	0.061	−0.004	0.597	0.042	0.006	0.136	0.106
L hippocampus	−0.005	0.627	0.040	0.004	0.213	0.111	0.016	0.060	0.148
R hippocampus	0.004	0.210	−0.102	−0.003	0.464	0.066	**0.025**	**0.028**	**0.174**
Total brain volume	−0.003	0.567	0.038	−0.001	0.384	0.063	0.007	0.109	0.103

*Note:* Bold results highlight significant results.

Abbreviations: DLPFC, dorsolateral prefrontal cortex; L, left; R, right; SDMT, Symbol Digits Modalities Test; TMT‐A, Trail Making Test A; TMT‐B, Trail Making Test B.

The number of *MVPA periods* did not improve the model fit for any outcome (Table 5). The number of periods in which *cognitive activity and MVPA* were present together (*cog activity PA periods*) was only associated with the immediate recall task. Periods in which *cognitive games and MVPA* co‐existed (*cog game PA periods*) provided the most associations with cognitive and volumetric variables. *Cog game PA periods* improved cognitive variable model fits for SDMT, TMT‐A and TMT‐B. In addition, greater *cog game PA periods* were associated with volumetric variables, improving model fits for the right dorsolateral prefrontal cortex, right hippocampal and total brain volumes. The addition of further control variables (Appendices A2 and A3) appeared to have the greatest impact on the number of outcomes associated with activities that included MVPA measures.

**TABLE 5:** Contribution of MVPA periods, cognitive activity and MVPA periods, and cognitive game and MVPA periods to cognitive and brain volume through a hierarchical model controlling for age, sex, and education.

	MVPA periods			Cog. activity PA periods			Cog. game PA periods		
	Δ adj. *R* ^2^	*p* of delta *R* ^2^	Beta	Δ adj. *R* ^2^	*p*	Beta	Δ adj. *R* ^2^	*p*	Beta
Cognitive performance									
SDMT	0.000	0.328	−0.081	−0.001	0.357	0.080	**0.026**	**0.024**	**0.179**
Immed. Recall	−0.005	0.187	0.109	**0.018**	**0.0499**	**0.169**	0.012	0.087	0.137
Delayed recall	0.001	0.377	−0.073	−0.006	0.735	0.030	0.001	0.300	0.083
Digit span backwards	0.002	0.435	−0.065	−0.006	0.967	0.004	−0.006	0.753	0.025
TMT‐A	0.002	0.418	0.068	−0.007	0.926	−0.008	**0.027**	**0.025**	**−0.183**
TMT‐B	0.006	0.772	0.024	0.005	0.178	−0.117	**0.030**	**0.018**	**−0.189**
Regions of interest									
L DLPFC	0.005	0.150	0.105	−0.004	0.715	0.028	0.008	0.107	0.114
R DLPFC	0.003	0.198	0.095	−0.001	0.361	0.071	**0.022**	**0.022**	**0.164**
L hippocampus	0.003	0.235	−0.098	−0.005	0.629	−0.042	0.009	0.120	0.125
R hippocampus	−0.006	0.824	0.019	0.001	0.279	0.094	**0.021**	**0.040**	**0.165**
Total brain volume	0.002	0.222	0.082	0.005	0.147	0.102	**0.042**	**0.001**	**0.217**

*Note:* Bold results highlight significant results.

Abbreviations: DLPFC, dorsolateral prefrontal cortex; L, left; R, right; SDMT, Symbol Digits Modalities Test; TMT‐A, Trail Making Test A; TMT‐B, Trail Making Test B.

## 4 Discussion

This study took an innovative approach to investigating how the lifestyle factors of physical activity and cognitive engagement relate to cognitive performance and regional gray matter volumes in older adults. The main findings were that engagement in cognitive activities is associated with better cognitive performance, and that engagement in physical activity was not independently associated with cognitive performance. Further, engagement in cognitive and physical activity with close temporal proximity to one another is associated with better brain structure. The fact that cognitive engagement was found to be associated with better cognitive performance is interesting and consistent with previous findings, but particularly noteworthy because this effect appears to be impacted by the type of cognitive activities engaged and only related to certain cognitive performance domains. The associations with brain structure were also noteworthy, as the results support the potential that the combination of cognitive and physical activities provides better conditions in which brain structure can be supported. As such, this research provides novel evidence of how cognitive and physical engagement may synergistically and uniquely be associated with higher cognitive or structural brain health.

The presence of physical activity within periods in which cognitive games were recorded did improve the number of associations with our outcomes, particularly in terms of regional gray matter volumes (see Table 5). The finding is consistent with the rationale that physical activity provides a rich neurotrophic environment, that sufficiently stimulating cognitive engagement could then convert into increased neuroproliferative, morphological and synaptogenic growth [19]. Although these findings do not provide causal evidence, it is consistent with emerging interventional evidence [2] that combining physical and cognitive activity may provide superior cognitive benefits to undertaking those activities in isolation.

The benefits of greater engagement in a cognitively‐rich lifestyle for cognitive performance benefits are supported by interventional approaches [47, 48]. Capturing this empirically within an observational design, however, provides insight into what might be important about habitual engagement for brain health. Our method of capturing cognitive engagement, through self‐report diaries, has previously been associated with cognitive outcomes [28] that are consistent with our findings (Table 4). Our observation that periods coded as containing cognitive games was associated with improved cognitive outcomes, more than broader cognitive engagement, may not be surprising. e.g., self‐reporting of engagement in Sudoku and similar puzzles has been positively associated with working memory, reasoning and episodic memory [10], and board and card game benefits to cognition linked to social interaction elements [11]. Although our diary approach is likely to capture broader information regarding the type of cognitive activities, our data cannot account for the intensity of engagement, nor distinguish levels of enjoyment or social interaction relating to these activities. Intuitively, these elements will impact the efficacy of cognitive engagement on cognitive performance. This could explain why the duration of cognitive activities (i.e. periods) was not strongly associated with cognitive performance, and why *cognitive games*, as captured here, were more commonly associated with cognitive performance if we assume that this measure is likely to encourage deeper engagement, enjoyment and socialization. Further, with minimal overlap, there were differences in the cognitive domains associated with the broader cognitive activity, and those coded as cognitive games. Thus, the tasks captured under the different codes likely represented different cognitive processes and consequently contribute in different ways to healthy brain aging. The optimal dosing of cognitive engagement is not well understood, but evidence on the contribution of the amount [49] as well as nature, degree of social engagement, and other attributes of the cognitive activity continue to emerge. Our findings that cognitive engagement was associated with better outcomes are nonetheless interesting and support global guidelines for healthy cognitive aging.

Physical activity did not appear to offer substantive associations in isolation. Long‐term engagement in physical activity has been unambiguously demonstrated to support brain health [13, 14]. However, we did not observe any evidence for this in this cross‐sectional investigation. It may be that our cross‐sectional measures do not reflect long‐term physical activity engagement, or that our measure of physical activity—a count of the number of time periods in which at least 15 min of MVPA was recorded—is not precise enough or representative of the dose of physical activity required to observe associations with cognitive and brain outcomes established by others. Further, when controlling for a greater number of variables, including physical health measures such as diabetic status, hypertension and body mass index, less outcomes were associated with activities that included the MVPA measures. This may indicate that a major role that physical activity plays is in the maintenance of a physical health environment in which the brain can maintain healthy cognition. Future research may be better placed to model meaningful dose parameters for associations between physical activity and brain health outcomes. The results of this study are also likely influenced by the various methodological choices. Whilst there are arbitrary elements to many of our threshold determinations (e.g., our selection of grouping activity within specific 3‐h time periods) many are supported by a mechanistic rationale. For example, there is a suggestion that 15 min of physical activity is an ideal dose [50] for supporting acute cognitive performance, which, if repeated over time, would align with principles of overload to promote positive adaptation.

The findings of this study have several potential implications. From a practical perspective, the findings suggest that thoughtful planning of activities throughout the day, whereby physical activity could be implemented within and surrounding activities that are more cognitively engaging, may be beneficial long‐term, supporting cognitive and brain reserves. At the least, this approach does not appear to do any harm to cognitive and brain health. Having the time and means to engage in both physical and cognitive activities however may depend on several factors including underlying health, financial resources, social support and more. Whilst this may not need to be intensive—for example, a brisk walk for 15 min followed by a card game of solitaire could have met the criteria that was used within this study—these barriers remain an important consideration in which guidelines and alternative options could assist with. More broadly, the findings of the study provide a consideration for how frameworks around cognitive and brain reserves are created. The differing associations between the single elements of cognitive activity, physical activities, and a combined metric with both cognitive and volumetric outcomes can contribute to understanding how these related, but separate, concepts of cognitive and brain reserves manifest. In these ways, further research into the potential implications of the current study is required.

Whilst this study provides important insights, several limitations have been discussed or need further acknowledgment. The cross‐sectional nature of this study prevents causal conclusions, but this study should help inform future RCT or longitudinal data that would be better placed to make these inferences. As highlighted in the methods, multiple analyses were conducted at the nominated alpha of 0.05. Whilst this was justified given the conservative nature of several measures, this approach is likely to inflate type 1 errors within the analyses run. As such, this provides a limitation that should be considered when interpreting the results. Further, some activity measures are likely to share variance and should be considered when interpreting the results holistically. Similarly, the activity measures are likely conflated with social interactions, and thus the use of the cognitive activity term used should be interpreted cautiously with this in mind. Finally, the self‐reported nature of the diaries, and the possibility of forgetting, may have limited the accuracy of the diary content recorded. For example, it is possible that the richness of the diaries related to certain cognitive domains [28], and so our diary method may have been effective in relating to cognitive performance not through reflecting activity, but through diversity in the depth of recorded entries. The short window in which participants were asked to fill out the diary—they could add to the diary in real‐time, and were encouraged to complete it daily—should however reduce the amount of data lost, or inflated, due to forgetting. This active participation could have also lead to changes in behavior over the week recorded, both for the activity diary and the physical activity monitoring, although the more passive nature of the physical activity recording may lessen that impact. For example, there is potential that the higher functioning individuals could have raised their cognitive activity further during the data collection period. Further research is needed to understand and account for these potential limitations.

## 5 Conclusion

This study provides novel evidence on how cognitive and physical engagement may synergistically be associated with higher cognitive and structural brain health. These positive associations are however limited and specific. Physical activity alone was not associated with the cognitive performance and regional gray matter volumetric outcomes in this study. By contrast, periods of reported cognitive activity and cognitive game activity, especially when conducted within periods that included MVPA, were commonly associated with cognitive performance and regional gray matter volumes. This study highlights the importance of improving our understanding of dose characteristics and the complexity of interaction that exists between lifestyle elements on our health.

## Conflicts of Interest

The authors declare no conflicts of interest.

## Data Availability

The de‐identified participant data that support the findings of this study are not publicly available but are available from the study committee upon reasonable request. The study was not pre‐registered.

## Appendix

Table A1

**TABLE A1:** Characteristics of participants between study subsamples.

Measure	Original PATH sample (wave 1)	MRI sample (at wave 4)	Current sample
Sample size	2550	275	155
Age, years (SD)	75.6 (1.5)	75.2 (1.4)	**75.1 (1.3)**
Education, years (SD)	14.1 (2.7)	14.3 (2.7)	14.3 (2.6)
BMI, kg.m^−2^ (SD)	26.69 (4.85)	26.34 (4.02)	26.20 (4.19)
Female, *n* (%)	1234 (44.4%)	116 (42.2%)	69 (44.5%)

*Note:* Bold results highlight significant results from the original PATH sample.

Abbreviation: BMI: Body mass index.

Table A2

**TABLE A2:** Contribution of activity diversity, and number of cognitive activity periods, or cognitive game periods to cognitive and brain volume through a hierarchical model controlling for age, sex, and education as well as APOE**e*4 genotype, hypertension, diabetes, smoking status, depression, alcohol consumption and body mass index.

	Activity diversity			Cognitive activity periods			Cognitive game periods		
	Δ adj. *R* ^2^	*p* of delta *R* ^2^	Beta	Δ adj. *R* ^2^	*p*	Beta	Δ adj. *R* ^2^	*p*	Beta
Cognitive performance									
SDMT	**0.019**	**0.037**	**0.164**	0.008	0.118	0.139	**0.050**	**0.002**	**0.245**
Immed. Recall	−0.003	0.424	0.070	−0.001	0.353	0.092	0.003	0.246	0.102
Delayed recall	−0.006	0.693	0.034	0.003	0.223	0.119	0.016	0.073	0.154
Digit span backwards	0.005	0.188	0.111	0.017	0.061	0.178	−0.005	0.639	0.040
TMT‐A	0.015	0.084	−0.154	−0.004	0.490	−0.070	**0.030**	**0.026**	**−0.196**
TMT‐B	0.001	0.275	−0.098	0.008	0.137	−0.145	**0.041**	**0.008**	**−0.223**
Regions of interest									
L DLPFC	−0.004	0.652	0.033	−0.005	0.782	−0.023	−0.001	0.386	0.064
R DLPFC	−0.003	0.480	0.053	−0.005	0.744	0.028	−0.001	0.392	0.064
L hippocampus	−0.007	0.892	0.012	−0.003	0.465	0.070	0.016	0.067	0.156
R hippocampus	**0.026**	**0.031**	**−0.181**	−0.006	0.678	−0.040	**0.022**	**0.041**	**0.172**
Total brain volume	−0.004	0.824	0.015	−0.002	0.431	0.061	−0.001	0.352	0.064

*Note:* Bold results highlight significant results.

Abbreviations: DLPFC, dorsolateral prefrontal cortex; L, left; R, right; SDMT, Symbol Digits Modalities Test; TMT‐A, Trail Making Test A; TMT‐B, Trail Making Test B.

Table A3

**TABLE A3:** Contribution of the number of MVPA periods, cognitive and MVPA periods, or cognitive game and MVPA periods to cognitive and brain volume through a hierarchical model controlling for age, sex, and education as well as APOE**e*4 genotype, hypertension, diabetes, smoking status, depression, alcohol consumption and body mass index.

	MVPA periods			Cog. activity PA periods			Cog. game PA periods		
	Δ adj. *R* ^2^	*p* of delta *R* ^2^	Beta	Δ adj. *R* ^2^	*p*	Beta	Δ adj. *R* ^2^	*p*	Beta
Cognitive performance									
SDMT	**0.018**	**0.042**	**−0.172**	−0.005	0.753	−0.029	0.008	0.130	0.123
Immed. Recall	−0.001	0.351	0.088	0.006	0.180	0.134	0.017	0.066	0.166
Delayed recall	0.000	0.330	−0.090	−0.007	0.937	0.008	−0.002	0.385	0.078
Digit span backwards	−0.004	0.540	−0.056	−0.007	0.981	−0.002	−0.006	0.804	−0.022
TMT‐A	0.001	0.301	0.099	−0.007	0.734	0.035	0.019	0.061	−0.172
TMT‐B	−0.003	0.430	0.074	−0.001	0.365	−0.089	**0.035**	**0.013**	**−0.217**
Regions of interest									
L DLPFC	0.010	0.086	0.135	−0.003	0.521	0.054	0.008	0.113	0.120
R DLPFC	0.010	0.093	0.135	0.004	0.182	0.114	**0.017**	**0.039**	**0.159**
L hippocampus	0.005	0.187	−0.121	−0.001	0.375	−0.087	0.006	0.175	0.119
R hippocampus	−0.006	0.764	−0.027	−0.007	0.832	0.021	0.013	0.090	0.147
Total brain volume	0.000	0.321	0.073	0.002	0.224	0.096	**0.027**	**0.007**	**0.190**

*Note:* Bold results highlight significant results.

Abbreviations: DLPFC, dorsolateral prefrontal cortex; L, left; R, right; SDMT, Symbol Digits Modalities Test; TMT‐A, Trail Making Test A; TMT‐B, Trail Making Test B.
